# Sustainable Recovery from Shocks: Policies and Partnerships for Fresh Produce Rescue and Environmental Impact Reduction

**DOI:** 10.3390/foods15030582

**Published:** 2026-02-05

**Authors:** Mariana T. Koutsopoulos, Luis F. Luna-Reyes, Christine T. Bozlak, Roni Neff, Tianhong Mu, Xiaobo Xue Romeiko, Zhijian Guo, Akiko S. Hosler, Stacy M. Pettigrew, Natasha Pernicka, Peter Crasto-Donnelly, Amy Klein, Beth J. Feingold

**Affiliations:** 1 Department of Environmental Health Sciences, College of Integrated Health Sciences, State University of New York (SUNY) at Albany, 1400 Washington Avenue, Albany, NY 12222, USA; 2 Rockefeller College of Public Affairs and Policy, State University of New York (SUNY) at Albany, 135 Western Avenue, Albany, NY 12203, USA; 3 Department of Health Policy, Management, and Behavior, College of Integrated Health Sciences, State University of New York (SUNY) at Albany, 1400 Washington Avenue, Albany, NY 12222, USA; 4 Department of Environmental Health & Engineering, Johns Hopkins Bloomberg School of Public Health, 615 North Wolfe Street, Baltimore, MD 21205, USA; 5 Department of Mathematics, State University of New York (SUNY) at Albany, 1400 Washington Avenue, Albany, NY 12222, USA; 6 Department of Epidemiology & Biostatistics, College of Integrated Health Sciences, State University of New York (SUNY) at Albany, 1400 Washington Avenue, Albany, NY 12222, USA; 7 Department of Allied Health Sciences, Albany College of Pharmacy and Health Sciences, 106 New Scotland Ave, Albany, NY 12208, USA; 8 Radix Ecological Sustainability Center, 153 Grand Street, Albany, NY 12202, USA; 9 The Food Pantries for the Capital District, 32 Essex Street, Albany, NY 12206, USA; 10 Capital Roots, 594 River Street, Troy, NY 12180, USA

**Keywords:** food policy, food donation, shock recovery, food waste, system dynamics

## Abstract

Food policies that respond to shocks and support nutritious diets for vulnerable populations can enhance resilience, support social equity, and reduce environmental damage. Using a simulation model, we evaluated the effectiveness of two food redistribution policies—Nourish New York, a program providing funds to food rescue organizations to purchase food directly from farmers, and the Food Donation and Food Scraps Recycling Law (an organics “waste ban”)—in response to a shock such as the COVID-19 pandemic. We assessed policy based on recovered food and life cycle carbon and water footprints over 10 years. Both policies improved produce donations during post-shock. The waste ban increased waste at feeding organizations; diverting unavoidable food waste to composting and anaerobic digestion mitigated its carbon footprint. Enhanced coordination and partnerships within the food redistribution network were crucial for ensuring that produce reached those in need, ultimately reducing long-term environmental impacts. Implementing multiple strategies that enhance recovery from farms and retail, while strengthening the organizational capacity of the food redistribution network, can simultaneously advance food security and environmental goals.

## 1. Introduction

Increasingly severe and frequent shocks—major short-term disturbances—threaten food and nutrition security by disrupting agriculture, the economy, society, and supply chains [1,2]. This study focuses on one shock-sensitive component of food systems: perishable fresh produce rescue—specifically the operations of food assistance organizations and their upstream sources (farms and retailers)—and evaluates the effectiveness of two redistribution policies (Nourish New York and the Food Donation and Food Scraps Recycling Law) in response to a shock, where limited shelf-life could rapidly translate into waste and reduced food access. While shocks can lead to immediate food insecurity, they also have long-term consequences for nutrition, health, and sustainability [3,4,5,6]. For instance, the COVID-19 pandemic significantly disrupted food access, particularly of low-income consumers [3,7,8,9], leading to changes in dietary patterns, food waste generation [7,8,10], and environmental impacts [11,12].

Food rescue—recovering and redistributing safe, quality food for human consumption [13], usually through donation—plays a critical role in meeting the needs of vulnerable populations during shocks [14,15,16,17]. At the same time, disruptions to food assistance organizations can hinder their ability to recover and redistribute perishable food, potentially increasing waste [18,19,20]. Food rescue can also contribute to environmental sustainability by preventing waste from over-production [21,22,23]. In addition, managing unavoidable food waste from food banks and food pantries through alternatives to landfilling (e.g., recycling pathways) may offset some environmental impacts associated with food recovery operations (e.g., packaging, transportation, refrigeration) [24,25,26,27,28].

Policies promoting food recovery and redistribution align with Sustainable Development Goals (SDGs) Target 12.3 (halving per capita food loss and waste by 2030) [29] and have gained attention in the U.S. and globally [30,31,32,33,34,35]. However, evidence remains limited on how these policies, such as Nourish New York and the Food Donation and Food Scraps Recycling Law, perform under shock conditions, when operational constraints and rapid decision-making can reshape both food access outcomes and waste management outcomes [30,36]. The recent literature also points to underreporting of co-benefits and trade-offs in food system policies, including strategies centered on the reduction in and redistribution of wasted food [36]. This gap is particularly important for perishable foods, where shelf-life losses and spoilage dynamics can shift the balance between recovery benefits and waste burdens during disruptions. We recognize that research across disciplines has examined how food policies and governance shape food access and surplus redistribution. Here, we complement that work by providing dynamic, policy-focused evidence that jointly examines quantitative estimates of how specific policies affect perishable food rescue operations, under shock conditions, while explicitly representing food decay and environmental trade-offs over time. The aim of this study was to examine how two specific policies to address food recovery and redistribution in New York respond to a short-term disturbance, and thus, contribute to the knowledge base on how best to create and implement these policies in order to withstand systemic shocks.

### 1.1. Conceptual Framework

Food systems are complex networks reliant on interconnected factors such as agriculture, weather, and economics [2,3,37,38]. This complexity can make it challenging to identify vulnerabilities and anticipation of intervention effects, complicating informed decision-making [2,37]. Systems thinking offers a holistic framework for understanding these interconnections. To analyze policy effects under disturbances, we employ system dynamics, which represents key accumulations (stocks), influencing actions (flows), and feedback loops to quantify system behavior over time and explore policy scenarios [39,40,41,42].

Resilience—the ability of a food system to maintain or improve outcomes after disturbances—is dynamic and evolves based on what is deemed desirable or detrimental over time [43,44]. System dynamics has been applied to assess vulnerability and resilience in contexts such as natural hazards [45,46,47], socio-ecological systems [2,43,48,49,50], and humanitarian food supply chains [51,52]. However, relatively few studies explicitly consider perishable foods and wasted food, and existing applications rarely capture the dynamics of food decay and shelf-life loss—processes that are central to understanding fresh produce recovery under disruption (2022) [51].

### 1.2. Study Focus and Contribution

We simulate policy effects under a system shock, focusing on fresh produce rescue in the New York State Capital Region—where long-standing partnerships with food assistance organizations enabled access to local operational information and supported model development. In this setting, surplus fresh produce is recovered from farms and retailers and routed through a network of intermediary distributors (e.g., food banks/food hubs) and food pantries to households. The Food Pantries Food Connect Map (https://map.thefoodpantries.org/) provides a helpful visualization of the regional food pantry network. Key place-based features include seasonal farm supply, a relatively consistent retail stream, capacity constraints (e.g., cold storage, volunteers, and staffing), transportation realities, and a set of available waste diversion pathways. We evaluated outcomes relevant to both food access and sustainability: recovered food, wasted food at food rescue organizations, and carbon and water footprints. The policies examined were the following:

- Nourish New York (Nourish NY): Provides funds to food emergency organizations to purchase fresh food directly from New York farmers [53]. Introduced in 2020 in response to the COVID-19 pandemic.
- Food Donation and Food Scraps Recycling Law (organics “waste ban”): Mandates large food waste generators to divert edible surplus to food aid organizations and recycle food scraps [54,55]. Effective January 2022, it aims to reduce waste and promote recycling.

These are active policies in New York State that represent two distinct mechanisms for improving fresh produce access during disruptions: (i) direct procurement from farms (Nourish NY) and (ii) diversion of edible surplus from large generators and increased organics diversion (organics waste ban).

We focus our contribution on a specific combination of elements that remains limited in prior work: we assess how real-world policies shape the redistribution of perishable foods during shocks while explicitly representing shelf-life dynamics and resulting trade-offs among recovery, waste, and environmental impacts over time. Existing research has primarily focused on agricultural production and a few commercial crops [56,57], with limited attention to food rescue and humanitarian supply chains. Although studies exists on waste management, supply chain resilience, and environmental impacts of food donation [18,28], they do not quantify how policies interact with disruption dynamics and perishability to generate co-benefits and trade-offs over time. Our study focuses on the following questions: (1) How do Nourish NY and the organics waste ban affect the quantity of fresh produce redistribution and perishability-related outcomes within the food rescue network—specifically redistributed quantity, average shelf-life (quality), and waste generation at feeding organizations—following a system shock and over time? (2) How do policy-driven changes in redistribution and waste management pathways affect life cycle carbon and water footprints, and what trade-offs emerge across fresh produce donation, waste reduction, and environmental outcomes?

By integrating policy design with the operational dynamics of fresh produce rescue, our analysis supports the development of environmentally sound policies [58,59,60,61,62,63]. This study is relevant to disaster resilience and response, public health, food system sustainability, regional planning, supply chain management, and social equity.

## 2. Methods

### 2.1. Approach

We generated a community-based system dynamics model and incorporated carbon and water footprints to simulate impacts relevant to food and nutrition security, sustainability, and environmental health. We used group model building, a well-established process to build these types of models [64,65], in collaboration with community organizations in the New York Capital Region [66]. This process involved workshops to capture participants’ knowledge and expertise into graphic representations, evaluate scenarios, and facilitate group discussions [64,67,68]. We incorporated model formulations and parametrized the model using a combination of local partner inputs (e.g., operational practices, handling constraints, and typical flows from farm and retail within the distribution network) and secondary data sources (further model details in Section 2.2). We performed simulations in Vensim DSS (10.1.5).

**Figure 1:**
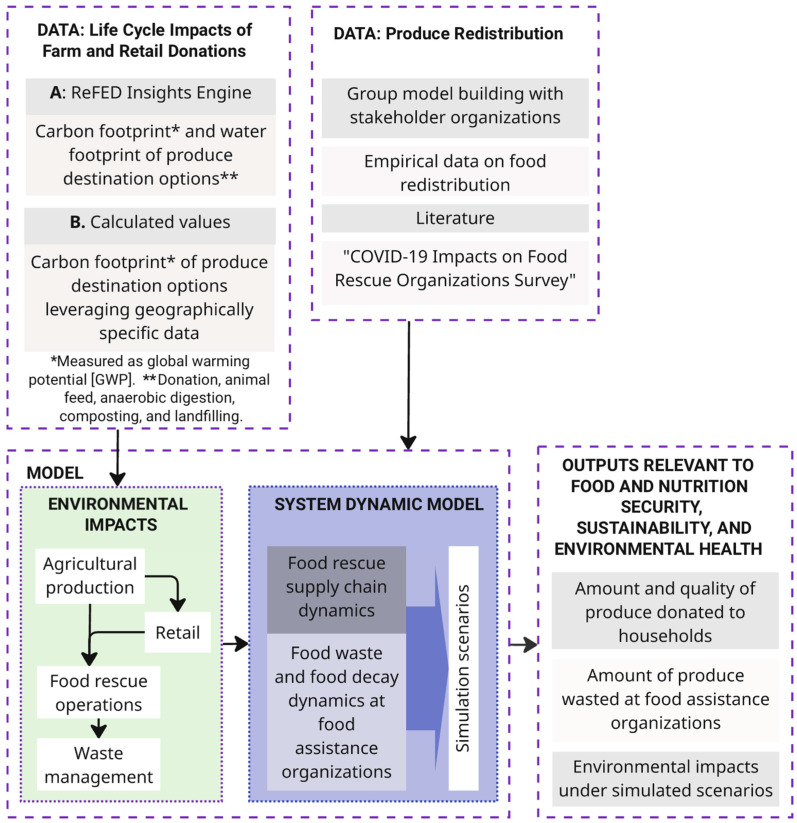
Modeling approach. Arrows indicate information/material flows.

We evaluated the success of policies based on their ability to sustain the provision of fresh produce to vulnerable populations, reduce waste, and minimize life cycle environmental impacts following disruptions. For life cycle environmental impacts, we used (a) open-source data [69] and (b) calculated values leveraging geographically specific data for the New York Capital Region. Figure 1 illustrates our approach.

### 2.2. The Model

#### 2.2.1. System Boundary, Scope, and Model Structure

*System boundary and rationale.* Our model represents fresh produce rescued by feeding organizations and the downstream handling of recovered food and unavoidable waste (e.g., spoilage at pantries and diversion to landfill, composting, and anaerobic digestion). We also account for the fact that not all donated produce is ultimately consumed. While the model includes surplus produce recovered from both farms and retailers—allowing us to represent differences in remaining shelf-life by source—we do not model avoided or generated waste occurring upstream (e.g., changes in retail waste generation or donation behavior). This boundary aligns with our study objective: evaluating how policies affect the performance and environmental outcomes of food rescue operations within the food rescue and feeding organization system. Accordingly, outcomes should be interpreted as net effects within the modeled system boundary, not as a full supply chain accounting.

*Model scope.* Building on Torres Arroyo et al. [66], the model represents fresh produce surpluses recovered from farms and retailers and redistributed through food assistance organizations to households, and it simulates wasted food generation at feeding organizations and households. Consistent with the system boundary above, the model does not include avoided or generated waste occurring elsewhere in the system (e.g., food wasted at retail but not donated). Within the model boundary, we estimate household waste and environmental emissions associated with redistribution based on whether recovered food reaches households, is recycled (e.g., composted or anaerobically digested), or is sent to landfill. The model incorporates supply chain dynamics described in the previous system dynamics literature [39], and dynamics related to waste at feeding organizations including waste rates, quality (average shelf-life), perceived food quality (a normalized variable that determines the fraction of wasted food), and quality losses (shelf-life losses) [66].

**Figure 2:**
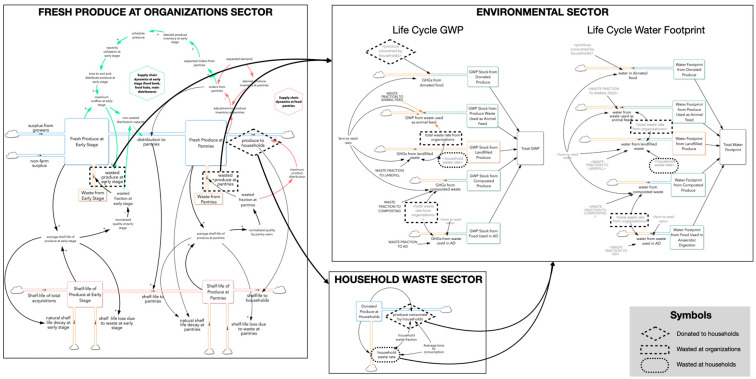
Overview of the model. The figure identifies connections among the three sectors represented in the model, stocks (boxes) and flows (double-lined arrows). Single-lined arrows indicate connections between variables or sectors.

*Model structure.* The model consists of the following sectors: Fresh Produce (fresh produce and its shelf-life); Household Waste; and Environment. Figure 2 presents a model overview. More detailed figures for each sector are available in the model documentation (Supplementary File S3) and a list of stocks and flows in the technical appendix (Supplementary File S1, Table S1).

Fresh Produce Sector: Encompasses fresh produce recovered and distributed by food assistance organizations and the food’s quality (using shelf-life as a proxy of food quality). Fresh produce is sourced from growers and retailers, accumulated at early-stage distributors like food banks and food hubs, and then distributed to food pantries for delivery to households. Retail produce is assumed to have lower shelf-life [66]. Food not donated to households gets wasted, and shelf-life is lost due to waste and natural decay. Further details on this sector, including the model-building process and more detailed definition of variables and parameters quality, can be found in Torres Arroyo et al. [66] and in the technical appendix (Supplementary File S1, Figure S1 and Table S1).

Household Waste Sector: Includes produce acquired by food pantry users, stored in households, and consumed or wasted. We assumed that produce from food pantries is consumed over one week, with 20% of the acquired produce ending up in landfills. According to studies on household waste in the U.S. [70] and donated surplus in Sweden [18], we expect that 20% to 25% of food donated to households may be wasted. This sector is included to avoid assuming that all redistributed produce is consumed; household waste does not respond endogenously to changes in household characteristics or storage conditions in the current model. We applied the following formulations (Equations (1)–(3)).

(1) $$\begin{array}{c} {Donated\;produce\;stock}_{households}(lbs.)\\ \;\;\;\;\;\;\;\;\;\;\;\;\;\;\;\;\;\;\;\;\;\;\;\;\;=\int Produce\;to\;households-Produce\;consumption-{Waste\;rate}_{households} \end{array}$$

(2) $$Produce\;consumption\;(lbs./week)=\frac{{Donated\;produce\;stock}_{households}}{Avg.\;time\;to\;consumption}\times (1-{Waste\;FR}_{households})$$

(3) $${Waste\;rate}_{households}\left(lbs./week\right)={Donated\;produce\;stock}_{households}\times \frac{{Waste\;FR}_{households}}{Avg.\;time\;to\;consumption}$$

Environmental Sector: Represents the life cycle carbon footprint (measured as global warming potential, GWP) and water footprint of donated food. These measures capture the greenhouse gas emissions and freshwater use associated with the production, transportation, storage, and waste management of recovered fresh produce. GWP is reported in carbon dioxide equivalents (CO_2_e), which expresses the warming impact of different greenhouse gases on a standardized scale by converting them to the amount of CO_2_ that would produce the same warming effect [71]. Environmental impacts consider the sources (farm and retail) and destination of fresh produce (households, composting, anaerobic digestion, and landfill). Equations (4)–(9) were used to integrate the life cycle GWP of fresh produce into the model based on its destination (*a–c* in Equations (4)–(9)). GWP is in kg CO_2_e for stocks and in kg CO_2_e/week for flows. GWP impact factors (GWP.IF) are in kg CO_2_e/lb. In Equations (7)–(9), Waste FR is the wasted fraction of produce at organizations (not donated) and sent to landfill or recycling options, and Ratio_farm:retail_ is the proportion of produce from farms with respect to that from retail. Waste_total produce rescue_ is in lbs. and includes all produce waste generated by food rescue organizations, and Waste_households_ is the produce waste generated at households. Formulations (Equations (4)–(9)) are as follows:

(a) Donated to households (Equations (4) and (5)):
  (4) $${GWP\;stock}_{donated}(kg\;{CO}_{2}e)=\int {GWP}_{donated}$$
  (5) $$\begin{array}{c} {GWP}_{donated}\left(kg\;{CO}_{2}e/week\right)\\ \;\;\;\;\;\;\;\;\;\;\;\;\;\;\;\;\;\;\;\;\;\;\;\;\;=\left({Ratio}_{farm:retail}\right)\times produce\;consumption\times {GWP.IF}_{donated\;farm\;produce}+(1\\ \;\;\;\;\;\;\;\;\;\;\;\;\;\;\;\;\;\;\;\;\;\;\;\;\;-{Ratio}_{farm:retail}\times {IF}_{donated\;retail\;produce}) \end{array}$$
(b) Recycled produce (Equations (6) and (7)–generic equations for produce diverted to animal feed, composting, and anaerobic digestion):
  (6) $${GWP\;stock}_{recycled}(kg\;{CO}_{2}e)=\int {GWP}_{recycled}$$
  (7) $$\begin{array}{c} {GWP}_{\;recycled}(kg\;{CO}_{2}e)\\ \;\;\;\;\;\;\;\;\;\;\;\;\;\;\;\;\;\;\;\;\;\;\;\;\;={(Ratio}_{farm:retail}\times {Waste}_{total\;produce\;rescue}\times {Waste\;FR}_{recycling})\times GWP.{IF}_{recycled\;farm\;produce}\\ \;\;\;\;\;\;\;\;\;\;\;\;\;\;\;\;\;\;\;\;\;\;\;\;\;+\left(1-{Ratio}_{farm:retail}\right)\times {Waste}_{total\;produce\;rescue}\times {Waste\;FR}_{recycling}\\ \;\;\;\;\;\;\;\;\;\;\;\;\;\;\;\;\;\;\;\;\;\;\;\;\;\times {GWP.IF}_{recycled\;retail\;produce} \end{array}$$
(c) Landfilled produce (Equations (8) and (9)):
  (8) $${GWP\;stock}_{landfilled\;produce}(kg\;{CO}_{2}e)=\int {GWP}_{landfilled\;produce}$$
  (9) $$\begin{array}{c} {GWP}_{landfilled\;produce}(kg\;{CO}_{2}e/week)\\ \;\;\;\;\;\;\;\;\;\;\;\;\;\;\;\;\;\;\;\;\;\;\;\;\;={(Ratio}_{farm:retail}\times {Waste}_{total\;produce\;rescue}\times {Waste\;FR}_{landfill})\\ \;\;\;\;\;\;\;\;\;\;\;\;\;\;\;\;\;\;\;\;\;\;\;\;\;\times {GWP.IF}_{landfilled\;farm\;produce}\\ \;\;\;\;\;\;\;\;\;\;\;\;\;\;\;\;\;\;\;\;\;\;\;\;\;+\left(1-{Ratio}_{farm:retail}\right)\times {Waste}_{total\;produce\;rescue}\times {Waste\;FR}_{landfill}\\ \;\;\;\;\;\;\;\;\;\;\;\;\;\;\;\;\;\;\;\;\;\;\;\;\;\times {GWP.IF}_{landfilled\;retail\;produce}\\ \;\;\;\;\;\;\;\;\;\;\;\;\;\;\;\;\;\;\;\;\;\;\;\;\;+{Waste}_{households}\times {GWP.IF}_{landfilled\;produce\;from\;organizations} \end{array}$$

The total GWP is the addition of GWP stocks. The structure of equations for water footprint mirrors that of the GWP formulations. Further details on GWP.IF values referred to in Equations (4)–(9) are in Section Life Cycle Impacts.

##### Life Cycle Impacts

We used environmental impact factors (values or coefficients) to estimate the life cycle environmental footprints of donated produce. We utilized factors for donation and waste management options. We used generic values from ReFED Insights Engine [69] to estimate the life cycle carbon footprint (global warming potential, GWP, in kg CO_2_e) and water footprint. We compared the GWP impact results with those we obtained based on the LCA (life cycle assessment) literature, including values available for our study area. Impact values used in the model are available in the technical appendix (Supplementary File S1, Tables S2 and S3).

***Generic emissions factors.*** ReFED Insights Engine—an open-access platform—provides state-level sector-specific estimates of food-related emissions, including both upstream emissions (from supply chain activities before food becomes waste) and downstream emissions (from waste management after disposal) [69,72,73]. It is grounded in real-world data and applications, integrating public and proprietary datasets, expert interviews, case studies, and industry research, providing a comprehensive picture of food waste in the U.S. [69,74]. Impact values consider life cycle emissions from food production to waste management, including transportation to destination, fugitive emissions, infrastructure, equipment use, and energy. The major assumptions of these data are summarized in the technical appendix (Supplementary File S1, Table S4) and discussed in Section 4.

***Calculated life cycle GWP for fresh produce redistributed in the New York Capital Region.*** These factors considered agricultural production [75,76,77,78,79,80], retailing [81], transportation [27,82], and waste management options: animal feed [72,83], composting [84], anaerobic digestion [85], and landfilling [86]. GWP life cycle impact factors (GWP.IFs) for each fresh produce category destination were calculated based on donation sources (farm and retail) and destination (e.g., donation, composting, landfill). Due to the lack of available data, GWP.IF_donation_ excludes transportation from food pantries to households. GWP.IF_animal feed_ accounts for avoided emissions from substituting corn in feed, using a 7:1 waste replacement ratio [72] and irrigated corn production [83]. GWP.IF_AD_ considers avoided emissions from the process [85], utilizing food waste for natural gas and electricity generation [87]. We used the value for food waste to anaerobic digestion with biogas used for electricity generation. GWP.IF_composting_ reflects the composting process [84]. GWP.IF_landfill_ includes emissions from produce landfilling based on the WARM model v.16 [86], accounting for energy recovery from operational landfill gas-to-energy projects in New York State [88], where most facilities convert waste to electricity, although the specific energy recovery method for several facilities is unknown. Calculations of GWP.IFs are provided in the technical appendix (Supplementary File S1, Table S7).

To incorporate agricultural emissions from donated produce, we used a GWP weighted average based on the weight fractions of produce types donated by farms and retailers in New York, along with their agricultural GWP. For retail donations, we accounted for storage impacts at U.S. perishables distribution centers and supermarkets [81], with donations consisting of 44.8% fruits and 55.2% vegetables. We assumed fresh produce is transported in refrigerated trucks powered by diesel. Transportation emissions were calculated based on travel distance from donors’ locations to feeding organizations in the New York Capital Region [27], using a factor of 0.07436 kg CO_2_e per 1 tkm (0.0000231 kgCO_2_e per 1 lb.-mile)—the freight equivalent to the transport of 1000 kg of food—from the GREET R&D Model [82]. Weight fractions are provided in the technical appendix (Supplementary File S1, Table S5).

### 2.3. Defining Disturbances and Key Outcomes Relevant to Resilience

We defined desirable and undesirable outcomes based on their contribution toward food and nutrition security, equitable food systems, sustainability, and environmental health goals. Outcome variables and goals are listed in the technical appendix (Supplementary File S1, Table S6). We evaluated recovery time after the shock, distinguishing between total recovery—achieving fresh produce distribution rates equal to or better than pre-shock levels—and partial recovery. To test for this recovery time, we explored the effect of increasing the severity of the shock, comparing a moderate vs. a severe shock (30% and 50% increased demand, 30% and 50% reduced supply, and 30% and 50% increased distribution times compared to the system at equilibrium).

To simulate the shock and organizational responses, we incorporated contextual information from food assistance organizations in the New York Capital Region during the first year of the COVID-19 pandemic. This information came from the literature and an online survey titled ‘COVID-19 Impacts on Organizations Involved in Produce Recovery and Redistribution in the New York Capital Region: 2020 to mid-2021’ (hereafter referred to as ‘COVID-19 Impacts on Food Rescue Organizations Survey’), co-designed by Capital Region FRESH academic and community partners, and administered via Qualtrics from February to June 2021. The survey gathered responses from 26 organizations, including farms, gleaners, food pantries, food hubs, food banks, and waste management organizations, focusing on changes experienced from March 2020 to June 2021 (see examples in Supplementary File S2). We conducted a qualitative analysis of responses, coding data and summarizing themes. Findings indicate that the COVID-19 shock prompted various changes and responses from food assistance organizations (a summary is available in Supplementary File S2).

### 2.4. Simulation Scenarios

We simulated a shock (baseline scenario) characterized by increased food demand, decreased food supply due to supply chain disruptions, and reduced capacity at organizations. At baseline, it was assumed that 60% of the produce that was not effectively donated (wasted) was diverted to animal feed, while 40% was sent to landfills, based on data from the regional food bank [27]. Scenarios tested policy implementation and their impacts under this shock (Table 1).

We examined the effects of implementing Nourish NY, which increases the supply of fresh produce from farms to main distributors (e.g., food banks, food hubs), and the organics waste ban, enhancing the flow from retailers. Potential increases in supply (i.e., 150% and 11% in Table 1) were based on diversion rates from the farm and retail sectors, respectively, in New York [89,90,91,92,93,94], as published by Torres Arroyo et al. [66].

We simulated increased coordination and partnerships (C&P) across organizations to improve distribution times (equations in Supplementary File S3). Based on the literature, the group model-building process, and the COVID-19 Impacts on Food Rescue Organizations Survey, we hypothesized that C&P would bolster resilience to shocks. We also tested a recycling policy (‘R’—included in S4, S8, and S9 in Table 1), assumed to divert half of landfilled waste to composting and half to anaerobic digestion.

In our model, the shock and policies are exogenous, while increased C&P are endogenous. Policies were implemented at year 1. The success of policies and increased C&P was assessed based on their ability to recover and sustain the provision of fresh produce to vulnerable populations while minimizing waste and environmental impacts over 1-year, 2-year, 5-year, and 10-year time horizons, reflecting short-to-long-term outcomes.

## 3. Results

In this Results section we compare nine scenarios (S) under a moderate shock that show (i) how policy and organizational interventions change fresh produce redistribution outcomes in the rescue network (donations, average shelf-life/quality, and waste) across time horizons, and (ii) how those changes translate into carbon and water footprint trade-offs.

### 3.1. Changes in Donations, Quality, and Waste of Fresh Produce at Food Assistance Organizations After the Shock

Section 3.1 compares how each intervention changes donations, average shelf-life (quality), and waste at food assistance organizations after the shock—outcomes that determine whether policy improves access without increasing spoilage-driven losses. Table 2 outlines these outcomes reported as cumulative percent changes relative to baseline, aggregating differences over a time horizon; accordingly, the results are most informative for comparing scenarios/policies and outcomes within the same time horizon (and across outcomes and scenarios), rather than interpreting the percentage magnitudes as standalone absolute numbers. Scenarios involving the recycling policy (S4, S8, and S9) are excluded from this table, as recycling is treated separately from produce redistribution, with no assumed feedback or rebound effects related to waste management of produce discarded by organizations.

Our findings show that the implementation of both Nourish NY (S1) and the organics waste ban (S2) after a shock positively impacted both the quantity and quality of produce redistribution, but the organics waste ban gradually increased waste at feeding organizations over time.

Coordination and partnerships (C&P) significantly improved the effectiveness of both redistribution policies, increasing fresh produce donations to the community and quality. Nourish NY alone (S1) had a minimal effect on donations in year 1, with only a 1% increase in quantity and quality from the baseline. However, with C&P (S5), donations rose by 21% and quality improved by 25%.

Nourish NY (S1) consistently reduced wasted produce over time. While initially increasing waste by 4% in year 1 with C&P (in S5), it ultimately led to less waste in the long term (see S1 vs. S5 in Table 2). The organics waste ban (S2) increased waste but became effective for both redistribution and waste reduction when combined with enhanced C&P (S6). Combining Nourish NY and the organics waste ban with enhanced C&P (S7) resulted in the best outcomes for increased produce donations and improved quality, and the second-best scenario for long-term waste reduction.

#### Recovery Time

The recovery time analysis answers how quickly each scenario restores produce distribution to pre-shock levels, distinguishing partial recovery from full recovery. This matters because speed of recovery is a key resilience metric: policies that eventually increase donations may still fall short if they do not restore access within the time window when households most need support.

Only Nourish NY (S1), the combination of Nourish NY with coordination and partnerships (C&P) (S5), and Nourish NY plus the organics waste ban and C&P (S7) achieved total recovery (produce distribution rates equal or better than pre-shock levels, defined in Section 2.3). After year 1 (week 65), these scenarios exceeded pre-shock produce distribution levels by 18%, 27%, and 38%, respectively. All scenarios reached partial recovery, with about 60% recovery in the first 2 weeks and 65% by week 13. After year 1 (week 65), the organics waste ban (S2) achieved 72% recovery, enhanced C&P (S3) reached 80%, and the organics waste ban combined with enhanced C&P (S6) reached 87%. Under the severe shock, it enabled Nourish NY (in S5) and the organics waste ban (in S7) to achieve full recovery, with S7 exceeding pre-shock levels at year 2.

### 3.2. Changes in Environmental Footprints After the Shock

Section 3.2 answers how the same interventions shift life cycle carbon and water footprints by changing (i) where produce is sourced (farm vs. retail) and (ii) where unavoidable waste is directed (landfill vs. composting/anaerobic digestion). This matters because the environmental performance of food rescue policies depends not only on how much food is redistributed, but also on perishability-related waste and the waste management pathways available to food assistance organizations. We report footprints using both ReFED factors and region-specific estimates to assess whether policy comparisons are sensitive to emissions assumptions for this case.

**Figure 3:**
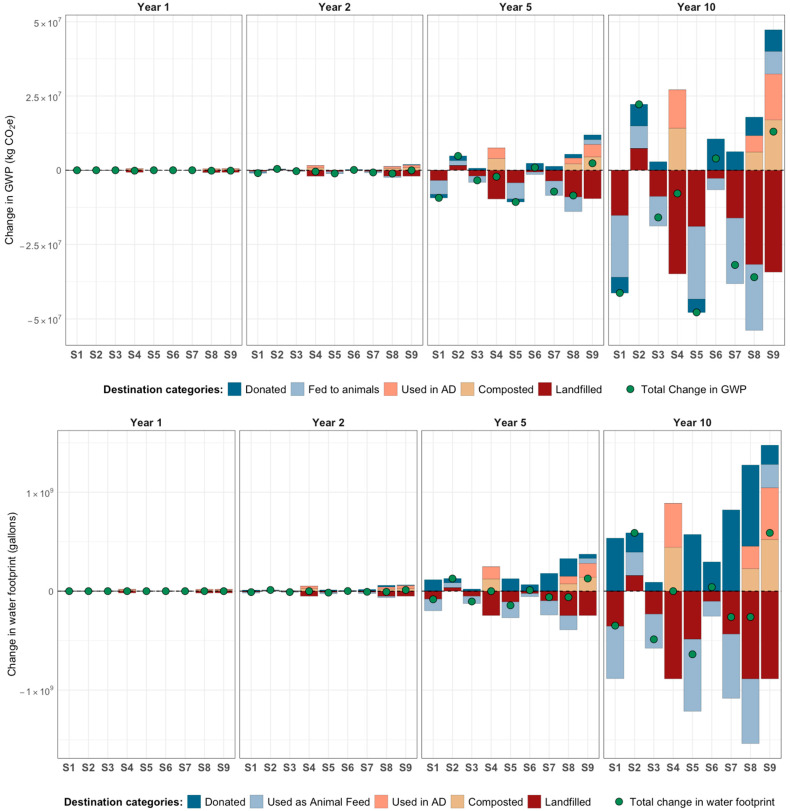
Cumulative change in carbon (top) and water (bottom) footprints from fresh produce redistribution: Results for nine scenarios (S) *using generic emission factors (from ReFED)*. Each scenario leads to increases or decreases in environmental footprints, driven by changes in food sourcing and waste destinations resulting from interventions. Scenarios represent intervention groups: redistribution policies (Nourish NY [NNY] and organic waste ban [WB]); organizational interventions (increased coordination and partnerships [C&P]); and recycling (diversion from landfills to anaerobic digestion and composting). Scenarios: S1: NNY; S2: WB; S3: C&P; S4: Recycling; S5: C&P + NNY; S6: C&P + WB; S7: C&P + NNY + WB; S8: C&P + NNY + WB + Recycling; S9: WB + Recycling.

**Figure 4:**
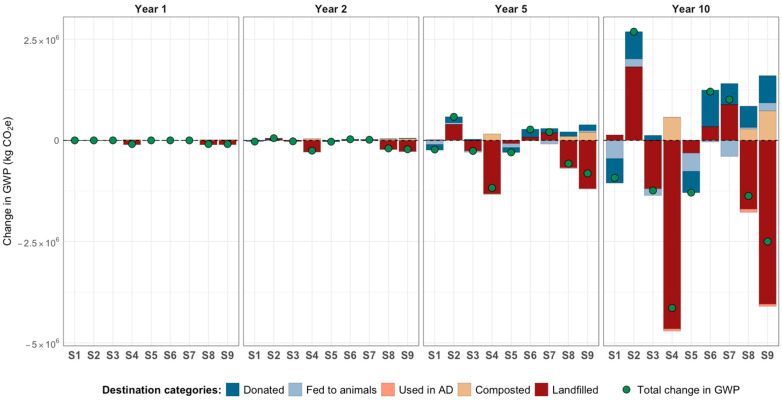
Cumulative change in carbon footprint from fresh produce redistribution: Results for nine scenarios *using calculated life cycle emissions factors for the New York Capital Region*. Each scenario leads to increases or decreases in environmental footprints, driven by changes in food sourcing and waste destinations resulting from interventions. Scenarios represent intervention groups: redistribution policies (Nourish NY [NNY] and organic waste ban [WB]); organizational interventions (increased coordination and partnerships [C&P]); and recycling (diversion from landfills to anaerobic digestion and composting). Scenarios: S1: NNY; S2: WB; S3: C&P; S4: Recycling; S5: C&P + NNY; S6: C&P + WB; S7: C&P + NNY + WB; S8: C&P + NNY + WB + Recycling; S9: WB + Recycling.

Figure 3 presents the water and carbon footprints across the nine scenarios (S) using life cycle emissions factors from ReFED. Figure 4 illustrates the carbon footprint (global warming potential, GWP) using calculated values for the New York Capital Region (described in Section Life Cycle Impacts).

*Nourish NY and the Organics Waste Ban (S1 and S2)*. Nourish NY was more effective than the organics waste ban in reducing the total carbon and water footprints, consistent across all time horizons and for both the ReFED and calculated GWP values. This was primarily due to avoided emissions from reductions in landfilled food compared to the waste ban, which increased emissions from donations and waste management.

*Combined redistribution policies and enhanced coordination and partnerships (S1 vs. S5 and S2 vs. S6).* Combining Nourish NY with enhanced C&P (S5) further reduced life cycle carbon and water footprints. This was confirmed for carbon footprints using both ReFED and calculated factors.

*Diverting produce away from landfills (S4, S8, and [S2 vs. S9]).* Diverting produce waste from food assistance organizations to composting and anaerobic digestion (S4) reduced the carbon footprint and neutralized the water footprint compared to the baseline (shock, no-policy). This result was consistent across simulations using ReFED data and regionally calculated life cycle GWP values. Implementing Nourish NY and the waste ban along with enhanced C&P and recycling (S8) also led to a reduction in both carbon and water footprints compared to the baseline, again supported by both sets of GWP data. In contrast, implementing the organics waste ban alone (S2) increased the carbon footprint. However, when wasted produce was diverted to anaerobic digestion and composting instead of landfilling (as in S9), the net carbon footprint of the organics waste ban was reduced (see S2 vs. S9). Notably, S9 resulted in net avoided GWP emissions when using region-specific values but not when using ReFED values, highlighting the importance of emissions assumption (later discussed in Section 4).

## 4. Discussion

We discuss the findings in relation to our research questions by interpreting how interventions shape (i) post-shock fresh produce rescue performance—including redistribution, recovery, average shelf-life (quality), and waste at feeding organizations—and (ii) the resulting life cycle environmental trade-offs, reflected in carbon and water footprints driven by changes in sourcing (farm vs. retail) and waste destinations. In doing so, we return to the manuscript’s motivation—limited dynamic, policy-focused evidence on perishable produce rescue under disruptions—and interpret findings and net effects within the modeled system boundary.

Shocks, such as the COVID-19 pandemic, can significantly disrupt supply chains, leading to delays and shortages in food aid delivery. These disruptions disproportionately impact under-resourced communities, increasing their risk of food insecurity due to income loss, price changes, and rising demand for food assistance [3,15,95,96,97]. Our model evaluated redistribution policies under such conditions by simulating sudden changes in demand for food assistance, food supply, and time needed to redistribute fresh produce by food rescue organizations.

While the model represents a single shock for modeling purposes, real-world systems increasingly face multiple, compounding disruptions—such as climate events, economic changes, and public health emergencies—alongside chronic stressors like infrastructure limitations and labor shortages. The impact of any given shock depends on its type, magnitude, timing, and local context.

Our analysis focused on the New York Capital Region, where the specific configuration of farms, retailers, food assistance organizations, and environmental pressures shapes both system vulnerabilities and the effectiveness of policy and organizational responses. Although these dynamics are place-based, the broader insights are transferable: investing in resilience—through strategic partnerships, coordinated efforts, and robust redistribution systems—can strengthen food security, improve environmental outcomes, and support faster, more cost-effective responses to future disruptions [98].

Although our analysis is grounded in the NYS Capital Region, the model is designed to be portable to other settings by distinguishing a reusable core structure from location-specific inputs. The reusable structure links (i) recoverable supply entering the redistribution network, (ii) redistribution through intermediary organizations, (iii) shelf-life decay and quality loss, (iv) waste generation at organizations and households, and (v) diversion pathways (e.g., landfill, composting, anaerobic digestion) with associated environmental accounting. By contrast, several inputs are place-based and would require recalibration in another region. These include the following: (1) baseline inflow volumes by source (farms vs. retail) and source-specific shelf-life/quality assumptions; (2) transportation distances and routing assumptions among key nodes (e.g., farm-to-food bank distances); (3) organizational capacity and handling parameters that shape throughput and spoilage, including cold storage, volunteer/staffing capacity for receiving/sorting/repacking, and acceptance/rejection behavior tied to perceived quality; and local waste destination and diversion options, including the availability and capacity of composting and/or anaerobic digestion and the resulting shares of waste routed to each pathway (see details in the technical appendix, Supplementary File S1, and the model documentation, Supplementary File S3). We expect the direction of the main qualitative insight to generalize, while the magnitude of impacts will vary with the local network structure, logistics distances, and diversion infrastructure.

*Effectiveness of Nourish NY and the organics waste ban:* Our analysis of redistribution policies showed that both Nourish NY and the organics waste ban increased fresh produce distributions post-shock. Nourish NY, which recovers fresh produce directly from farms at peak shelf-life, was considerably more effective in reducing food waste and life cycle environmental footprints than the organics waste ban, which recovers lower shelf-life produce from retail [66]. Importantly, these findings reflect net effects within the modeled boundaries, i.e., changes in waste generation and diversion among food rescue and feeding organizations, and do not capture potential upstream changes in retail waste generation or donation behavior.

Within this boundary, the organics waste ban increased waste over time, in part because lower shelf-life retail produce raises the likelihood of spoilage after donation. At the broader system level, however, this policy may still reduce total waste by diverting material from retail that would have otherwise been landfilled; those avoided retail impacts are outside the current model scope and therefore are not reflected in our net estimates. Additionally, retail produce constitutes a relatively larger and more consistent supply of fresh fruits and vegetables to food assistance organizations than produce sourced from local farms, which has implications for policy design when reliability of supply is prioritized alongside waste and environmental outcomes.

Extending the boundary upstream in future work would allow the model to endogenize retail (e.g., shifts in discard rates, sorting effort, diversion compliance, and donation participation), therefore enabling a fuller accounting of system-level benefits and burdens associated with the organics waste ban or other policies.

This comparison extends prior work that evaluates food donation and waste management options [18,28] by showing how policy design interacts with food perishability (shelf-life) under disruptions to produce different waste and environmental footprint trajectories over time.

*The role of coordination and partnerships:* Coordination and partnerships (C&P) enhanced policy effectiveness by enabling quicker responses to rising food aid demand, improving the quality of donated produce, and reducing both waste and environmental footprints. The combination of policies—Nourish NY and the organics waste ban—alongside strengthened C&P led to the largest gains in fresh produce donations, quality, and waste reduction by year 5. These improvements were driven by increased donations to organizations, a higher proportion of top-quality produce through Nourish NY, and, critically, by improved organizational capacity to manage and distribute fresh produce efficiently—resulting in quicker distribution and reduced spoilage. These findings underscore the value of strengthening organizational capacity by supporting communication channels and partnerships across food rescue organizations, farmers, retailers, and government agencies. This is especially critical during periods of heightened demand, when operational challenges, such as reduced staffing and increased administrative burdens, intensify. Community partner feedback also emphasized the need for dedicated staff funding to sustain coordination efforts, noting that while funders cover program costs like equipment and materials, they are less likely to support staffing expenses. These dynamics align with resilience perspectives that emphasize adaptive capacity and coordination under stress [43,44] and help explain why policy effectiveness depends on organizational throughput constraints in shock conditions. On the other hand, enhanced C&P alone were not sufficient to offset the environmental footprint associated with implementing the organics waste ban. The results point to the need for a combination of strategies—not only those that build organizational capacity, but also those that divert food from landfills through methods like anaerobic digestion and composting.

*Environmental impacts of recycling:* The high perishability of fresh produce can lead to its waste before reaching consumers. However, an efficient system that recycles produce waste into useful products like animal feed, energy, and fertilizers can contribute to improved environmental outcomes. Our model indicated that diverting produce from landfills to composting and anaerobic digestion can offset the carbon and water footprints of donated produce, including emissions from agricultural production, cold storage, and transportation. This emphasizes the importance of incorporating circularity into food rescue systems, prioritizing resource reuse and nutrient recycling, instead of relying on the linear “make–use–dispose” model [99,100].

These findings are consistent with circularity-oriented policy rationales in food waste governance and with policies such as New York’s organics waste ban and a similar legislation in California (in Senate Bill 1383) that promote diversion and resource recovery [34,99,100,101]. At the same time, our model does not include feedback or rebound effects from recycling (e.g., behavioral or system responses that could change waste generation), which prior research in other sectors suggests could reduce net benefits [102,103]. Future research should incorporate these complex feedback processes to better understand the environmental effectiveness of recycling policies under real-world implementation dynamics.

*Utilizing ReFED and calculated GWP impact factors:* We conducted model simulations using two sets of life cycle carbon footprint estimates: (a) GWP values from the ReFED Insights Engine, and (b) values calculated specifically for our study region. While the absolute life cycle carbon footprint results differed between the two sources, both approaches led to consistent conclusions about policy effectiveness and the impact of recycling, with one exception: the scenario involving implementation of the organics waste ban with all wasted food diverted equally to anaerobic digestion and composting. In this case, using ReFED values resulted in a net increase in carbon emissions over time compared to the baseline, whereas regional values indicated a net decrease. This discrepancy stems from substantial differences in GWP impact factors for retail-sourced food waste recycled via anaerobic digestion and composting. In this scenario, all food is sourced from retail (through the organics waste ban’s implementation) and sent through recycling pathways that, under ReFED estimates, carry high emission burdens. In contrast, our region-specific values for these same processes are significantly lower (see Supplementary File S1, Tables S2 and S3). Unlike other scenarios, this one does not include additional interventions—such as sourcing from local farms through Nourish NY or enhanced distribution capacity—that might otherwise reduce waste volumes or shift waste toward lower-emissions pathways. Additionally, it is important to highlight that this same scenario had a lower net carbon footprint compared to implementing the organics waste ban alone in simulations using both ReFED and region-specific values. Overall, our findings suggest that ReFED impact factors can serve as a useful proxy for similar U.S.-based analyses when region-specific data are not available. However, ReFED’s impact factors for retail produce and recycling pathways may, in some cases, overestimate emissions compared to localized estimates. When possible, incorporating region-specific data—particularly for commonly used waste management methods—can improve accuracy and better reflect the local context, including factors such as types of food donated, fuel used for transportation, and travel distances from food sources to distribution points. This sensitivity to emissions assumptions reinforces that policy comparisons can be robust in direction yet contingent in magnitude, underscoring the value of transparent impact factor selection for decision-relevant environmental assessment.

In summary, the model clarifies how perishable produce rescue under shock conditions depends on both policy design (farm procurement vs. retail diversion) and operational capacity (coordination/partnerships) to preserve shelf-life and reduce spoilage-related waste. It also shows that environmental outcomes are shaped not only by recovery volumes but by waste destinations and the availability of circular pathways. Together, these findings respond to the research gap posed in the Introduction by providing a dynamic, policy-focused account of co-benefits and trade-offs over time within the modeled boundary.

### Strengths and Limitations

This study builds on the complexity of food system shocks by simulating the combined effect of redistribution policies during crisis conditions. The model revealed how coordinated interventions can influence key outcomes such as food donations, waste reduction, and environmental emissions. By incorporating aspects of food perishability—including shelf-life, non-linear decay dynamics, and waste rates—alongside distribution times, organizational capacity, and shifting demand, it captured time-sensitive dynamics that influence system performance under stress. Developed in collaboration with community partners, the model reflects the lived realities of food rescue operations and is grounded in both academic insight and practical expertise. It also draws on diverse and complementary data sources—including ReFED, life cycle assessment data, and empirical evidence from food redistribution efforts—which enabled context-specific assumptions and enhanced the model’s relevance to real-world planning and policy design.

The model also has some limitations that can be addressed in future research. It did not account for potential rebound effects of donations or recycling, which may diminish environmental benefits. On the one hand, previous studies indicate that donations are still favorable compared to anaerobic digestion despite these effects [18]. However, research on rebound effects in other sectors [102,103] argues that recycling introduces new actors, connections, and flows, potentially increasing waste and environmental emissions. These factors can be considered in future research. Additionally, we did not assess the impact of donated food on household waste, nor emissions from transporting fresh produce from food pantries to households. While including a household sector in our model prevents the unrealistic assumption that all redistributed produce is consumed, it is treated as static in the current model. Future work could refine this sector by endogenizing household waste dynamics, such as cold storage availability, household size, and perceived quality—and allowing the proportion of wasted food at home to vary over time and across scenarios. Future modeling may also consider practices like freezing and cooking to better understand the impacts of food donation. Other relevant aspects include the seasonality of redistributed produce, food type diversity, and economic costs of achieving zero waste across the food rescue system. Costs associated with policy implementation and social impacts were also not included. Though the environmental benefits of donations, as noted above, are likely to remain despite a rebound effect, economic savings might not [18,34]. Future research should explore the cost–benefit analysis of policies like Nourish NY and the organics waste ban and their potential to strengthen the communities they serve. As a final remark, we emphasize that navigating priorities within interconnected systems is essential, as strategies that advance food and nutrition security may not always align with those that maximize environmental benefits. Recognizing that not all interventions deliver equal co-benefits, decision-makers must carefully evaluate trade-offs to effectively balance competing goals and promote more holistic, sustainable outcomes.

## Figures and Tables

**Table 1:** Simulation scenarios (S).

Scenarios (S)	Farm Supply	Retail Supply	Distribution Time, Main Distributors	Distribution Time, Food Pantries	Destination of Waste *
S1: Nourish NY	base +150%	base	base	base	base
S2: Organics waste ban (WB)	base	base +11%	base	base	base
S3: Increased coordination and partnerships (C&P)	base	base	base −20%	base −20%	base
S4: Recycling (R)	base	base	base	base	0% landfilled
S5: C&P + Nourish NY	base +150%	base	base −20%	base −20%	base
S6: C&P + WB	base	base +11%	base −20%	base −20%	base
S7: C&P+ Nourish NY + WB	base +150%	base +11%	base −20%	base −20%	base
S8: C&P + Nourish NY + WB + R	base +150%	base +11%	base −20%	base −20%	0% landfilled
S9: WB + R	base	base +11%	base	base	0% landfilled

Baseline (base) values: Initial values of farm and retail supply, distribution times, and wasted produce to specific destinations under a moderate shock. Values at equilibrium (no shock, no policies): demand = 160,000 lbs./week; farm supply = 22,500 lbs./week; retail supply = 127,500 lbs./week; distribution time_main distributors_ = 2.5 weeks; distribution time_food pantries_ = 1.8 weeks. * Destination of waste at baseline: 40% landfill, 60% animal feed, and 0% recycling. Under a recycling policy (R), wasted food is diverted from landfill (0% landfilled produce) to composting (50%) and anaerobic digestion (50%). Grey background highlights change from baseline values.

**Table 2:** Cumulative changes * in fresh produce surplus redistribution by food assistance organizations following a shock and the implementation of state policies (Nourish NY and organics waste ban) and organizational interventions (increased coordination and partnerships across organizations [C&P]), compared to baseline.

	Outcome Variables											
Scenarios	Produce Donations (Cumulative %)				Quality of Fresh Produce (Cumulative %)				Wasted Produce (Cumulative %)			
	Year 1	Year 2	Year 5	Year 10	Year 1	Year 2	Year 5	Year 10	Year 1	Year 2	Year 5	Year 10
S1: Nourish NY (NNY)	1	78	311	700	1	177	706	1587	−10	−55	−191	−416
S2: Organics waste ban (WB)	0	8	33	73	0	3	13	28	0	22	88	200
S3: C&P	20	40	99	197	23	77	238	507	16	−17	−117	−283
S5: C&P + NNY	21	111	380	830	25	300	1127	2505	4	−62	−259	−587
S6: C&P + WB	20	50	141	291	23	83	261	559	16	1	−44	−120
S7: C&P + NNY + WB	21	128	448	982	25	311	1171	2603	3	−55	−230	−522

* Cumulative changes (%) reflect how each scenario deviates from the baseline (shock with no policies or interventions). Color coding: *Blue* indicates relatively more desirable changes, whereas *red* represents relatively fewer desirable ones. These visual cues show whether changes align with predefined goals: increased redistribution of fresh produce surplus, improved produce quality, and reduced waste.

## Data Availability

The original contributions presented in this study are included in the article/Supplementary Materials. Further inquiries can be directed to the corresponding authors.

## Supplementary Materials

The following supporting information can be downloaded at: https://www.mdpi.com/article/10.3390/foods15030582/s1. Supplementary File S1: Technical appendix. Supplementary File S2: COVID-19 Impacts on Food Rescue Organizations Survey: Main Findings and Sample Questions Supplementary File S3: Model documentation and Model Overview (contains all equations needed to replicate simulations).

## Glossary

Food rescue: Collection of safe, edible surplus food—typically from farms, grocery stores, restaurants, and institutions—for redistribution to people through food banks, pantries, shelters, or other charitable organizations. Global warming potential (GWP): Metric comparing the ability of different greenhouse gases (GHGs) to trap heat in the atmosphere and contribute to global warming over a specific time horizon. Life cycle assessment (LCA): Systematic method to quantify environmental impacts across a product’s life cycle—from raw material extraction through production and use to end-of-life (recycling, recovery, or disposal).

## Abbreviations

The following abbreviations are used in this manuscript:

AD	Anaerobic digestion
C&P	Coordination and partnerships across the food rescue organizations’ network
CO_2_e	Carbon dioxide equivalent
GHGs	Greenhouse gases
GWP	Global warming potential
GWP.IFs	Global warming potential life cycle impact factors
LCA	Life cycle assessment
Nourish NY/NNY	Nourish New York (New York State program funding purchases from NY farms for redistribution)
