# Supplementary material for: Sustainable Recovery from Shocks: Policies and Partnerships for Fresh Produce Rescue and Environmental Impact Reduction
Source: Foods. 2026 Feb 5;15(3):582. doi: 10.3390/foods15030582 (PMC12897177; doi:10.3390/foods15030582)
Supplement: Supplementary file 1 [file foods-15-00582-s001.zip › Supplementary File S1_Technical_Appendix-corrected.pdf]

Supplemental Information 1: Technical Appendix

Content:

- Figure S1. Fresh produce sector (diagram).
- **Table S1.** Stocks and flows of food, shelf-life, and environmental footprints.
- **Table S2.** Life cycle emissions factors of produce surplus destinations.
- **Table S3.** Calculated life cycle emissions factors of fresh produce redistributed in the New York Capital Region.
- **Table S4**. Major assumptions of ReFED impact factors.
- **Table S5.** Weight fractions of produce types donated by the farm and retail sectors in New York.
- **Table S6.** List of outcome variables, units, and associated goals.
- **Table S7.** GWP life cycle impact factor (IF) calculations for fresh produce distributed by food assistance organizations based on donation sources (farm and retail) and destination (donation, animal feed, anaerobic digestion, composting, and landfill).

Figure S1. Fresh Produce Sector

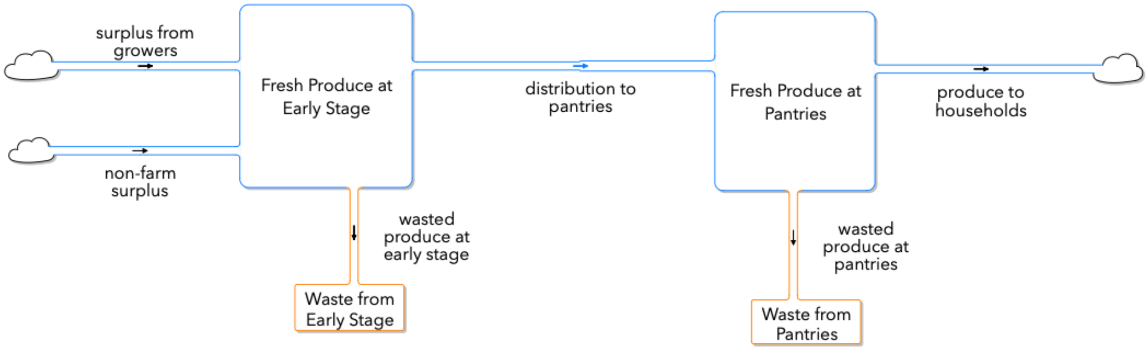

a.

b.

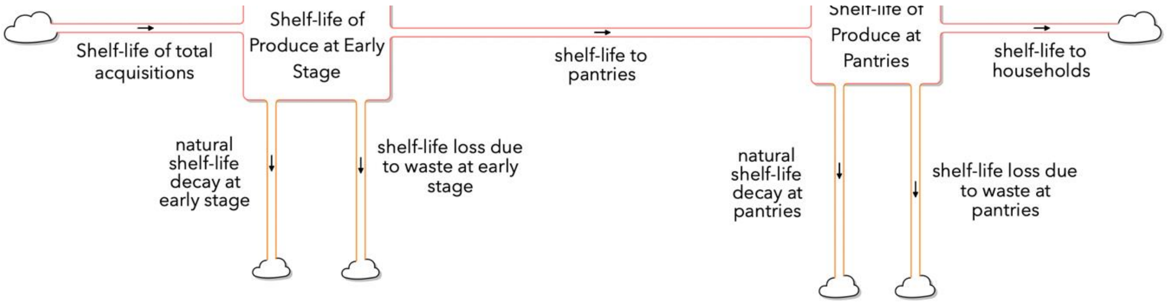

(***a***) *Fresh produce flows in the Fresh Produce Sector.* Units: lbs. (***b***) *Shelf-life flows in the Fresh Produce Sector*. Units: shelf-life/lbs. Boxes are stocks of fresh produce and shelf-life, in a and b, respectively.

The *Fresh Produce Sector* includes produce recovered and distributed by food assistance organizations and its shelf-life aggregated in two stages; major distributors (including food banks and food hubs) are the first stage, and food pantries the second stage. Fresh produce is sourced from growers and retailers (“non-farms”) and accumulated at the “early stage” (main distributors such as food banks and food hubs), then distributed to food pantries, which ultimately deliver it to households. Food not donated to households gets wasted.

Produce shelf-life is accumulated and distributed to food pantries and households, but it is also lost across the system due to natural food decay and waste. We used shelf-life as a proxy for quality, modeling it as a *co-flow*. A co-flow represents the simultaneous increase or decrease in two or more stocks in a system (in this case, fresh produce and its shelf-life), indicating a mutually reinforcing relationship between them or alignment in the same direction.

Table S1. Stocks and flows of food, shelf-life, and environmental footprints.

**Stocks**	*Food stocks*<br>(Units: lbs. of fresh produce)	- Fresh produce at early stage*.<br>- Fresh produce at food pantries.<br>- Fresh produce in households.
	Shelf-life stocks<br>(Units: weeks of shelf-life)	- Shelf-life of produce at early stage*.<br>- Shelf-life of produce at food pantries.
	GHG emissions stocks<br>(Units: kg CO_2e)	- Accumulated GHG emissions from diversion to animal feed.<br>- Accumulated GHG emissions from produce diverted to landfill.<br>- Accumulated GHG emissions from donated food.
	Water footprint stocks<br>(Units: gallons of water)	- Accumulated water footprint from diversion to animal feed.<br>- Accumulated water footprint from produce diverted to landfill.<br>- Accumulated water footprint from donated food.
**Flows**	*Food inflows*<br>(Units: lbs. of produce/week)	- Fresh produce from growers (*i.e.,* farms, orchards).<br>- Fresh produce from retail donors (*i.e.,* supermarkets, wholesalers, grocery stores).
	Shelf-life inflows<br>(Units: weeks of shelf-life/week)	- Shelf-life of fresh produce from growers.<br>- Shelf-life of fresh produce from retail donors.
	Food outflows<br>(Units: lbs. of produce/week)	- Produce distribution to households.<br>- Produce consumed by households.<br>- Wasted produce at feeding organizations.<br>- Wasted produce at households**.
	Shelf-life outflows<br>(Units: weeks of shelf-life/week)	- Shelf-life loss due to waste.<br>- Natural shelf-life decay.<br>- Shelf-life of the food distributed to households.
	GHG emissions flows<br>(Units: kg CO_2e/week)	- GHG emissions from donated produce.<br>- GHG emissions from use as animal feed.<br>- GHG emissions from anaerobically digested produce.<br>- GHG emissions from composted produce.<br>- GHG emissions from produce diverted to landfill.
	Water footprint<br>(Units: gallons of water/week)	- Water footprint from donated produce.<br>- Water footprint from use as animal feed.<br>- Water footprint from anaerobically digested produce.<br>- Water footprint from composted produce.<br>- Water footprint from produce diverted to landfill.

*Early stage = aggregated main distributors, food banks, and food hubs.
**Assuming constant household waste (20%) and average time to consumption (1 week).

Table S2. Life cycle emissions factors of produce surplus destinations.

Sector	Destination of wasted produce	Carbon footprint (kg CO2e/lbs. produce)	Water footprint (gallons/lbs. produce)
Farm	Anaerobic Digestion	0.0749	16.124
	Animal Feed	0.0616	16.124
	Composting	0.1217	16.124
	Donation	0.0584	8.062
	Landfill	0.2422	16.124
Retail	Anaerobic Digestion	0.6699	20.122
	Animal Feed	0.6667	20.122
	Composting	0.7276	20.122
	Donations	0.3620	10.061
	Landfill	0.8761	20.122

Data source: ReFED Insights Engine - Impact Calculator.

Table S3. Calculated life cycle emissions factors of fresh produce redistributed in the New York Capital Region.

Sector	Destination of wasted produce	Carbon footprint (kg CO2e/lbs. produce)
Farm	Anaerobic Digestion	-0.0227
	Animal Feed	-0.0051
	Composting	0.0206
	Donation	0.0099
	Landfill	0.1407
Retail	Anaerobic Digestion	0.0021
	Animal Feed	0.0197
	Composting	0.0455
	Donations	0.0324
	Landfill	0.1678

Table S4. Major assumptions of ReFED impact factors.

Impact Factor (IF)	Major assumptions
IF $_{Donation}$	• Food recovery will avoid impacts from food production.<br>• Not all donated food is suitable for human consumption. Food not suitable for human consumption will be landfilled. Each unit of food donated will result in 0.5 units less of food production required to meet the demand for food for human consumption (M. Ringland, personal communication, Oct. 2, 2023).<br>• Food recovery will avoid the demand for the same food category that would have been purchased by people receiving the food donation.<br>• The impacts of logistics are the same as those of retail centers.
IF $_{Animal\ feed}$	• Includes the transportation of waste to livestock farms, the transportation of this feed, and a feed offset assuming the replacement of feed-quality corn production.
IF $_{Anaerobic\ digestion}$	• Energy production will be avoided due to recovered biogas to energy and the generation of digestate, which will avoid NPK fertilizers.
IF $_{Composting}$	• Application of compost as fertilizer will avoid the use of NPK fertilizers and prevent soil degradation, improving long-term productivity.
IF $_{Landfill}$	• Avoided emissions due to landfill gas recovery to energy and landfill carbon storage.

Source: Corona et al. (2020).

Corona, A., Ernstoff, A., Segato, C. & Zgola, M. (2020). Greenhouse Gas Emissions of Food Waste: Methodology. https://refed.org/downloads/quantis-ghg-methodology-vfinal-2020-11-03.pdf.

Table S5. Weight fractions of produce types donated by the farm and retail sectors in New York.

Produce category	Item	Farm fraction	Retail fraction
Fruit	apples	0.616	0.095
Fruit	apricots	0.000	0.001
Fruit	avocados	0.000	0.053
Fruit	bananas	0.000	0.045
Fruit	grapes	0.000	0.027
Fruit	honeydew melons	0.000	0.007
Fruit	lemons	0.000	0.004
Fruit	mangoes	0.000	0.011
Fruit	orange	0.000	0.024
Fruit	peaches and nectarines	0.003	0.010
Fruit	pears	0.004	0.011
Fruit	pineapple	0.000	0.038
Fruit	strawberries	0.000	0.032
Vegetables	asparagus	0.000	0.009
Vegetables	bell peppers	0.000	0.020
Vegetables	broccoli	0.000	0.007
Vegetables	cabbage	0.093	0.006
Vegetables	carrots	0.000	0.015
Vegetables	cauliflower	0.000	0.009
Vegetables	celery	0.000	0.007
Vegetables	cucumbers	0.000	0.026
Vegetables	green bean	0.009	0.0086
Vegetables	squash	0.013	0.0153
Vegetables	pumpkin	0.004	0.0055
Vegetables	head lettuce	0.000	0.020
Vegetables	onions	0.068	0.021
Vegetables	peas	0.0003	0.0021
Vegetables	potatoes	0.123	0.066
Vegetables	radishes	0.000	0.004
Vegetables	romaine and leaf lettuce	0.000	0.020
Vegetables	spinach	0.000	0.002
Vegetables	sweet corn	0.043	0.003
Vegetables	tomatoes fresh	0.020	0.047

Example: The GWP of apples from farms is calculated as GWP_{apples} (CO_2e/kg) multiplied by the weight fraction of apples in total farm donations.

Table S6. List of outcome variables, units, and associated goals.

Outcome variables	Units	Goal associated with the outcome
Produce to households	lbs./week	Food Security, Nutrition Security, Equitable Food Systems
Waste rate	lbs./week	Food Security, Environmental Health, Sustainability
Quality of produce distributed to households	(weeks of shelf-life/lbs.)/week	Nutrition Security and Equitable Food Systems
Total global warming potential (GWP)	Kg CO_2e	Environmental Health, Sustainability
Total water footprint	Gallons of water	Environmental Health, Sustainability
GWP from produce donated to households	Kg CO_2e	Environmental Health, Sustainability
GWP from produce diverted to recycling options (animal feed, composting, and anaerobic digestion)	Kg CO_2e	Environmental Health, Sustainability
GWP from food diverted to landfills	Kg CO_2e	Environmental Health, Sustainability
Water footprint from produce donated to households	Gallons of water	Environmental Health, Sustainability
Water footprint from produce diverted to recycling options (animal feed, composting, and anaerobic digestion)	Gallons of water	Environmental Health, Sustainability
Water footprint from food diverted to landfills	Gallons of water	Environmental Health, Sustainability

Table S7. GWP life cycle impact factor (GWP.IF) calculations for fresh produce distributed by food assistance organizations based on donation sources (farm and retail) and destination (donation, animal feed, anaerobic digestion, composting, and landfill).

Factor	Farm Produce Donations	Retail Produce Donations
$GWP.IF_{donation}$	$-GWP_{agriculture-adj.farm}$ $+ GWP_{rescue\ transportation}$	$-GWP_{agriculture-adj.retail} + GWP_{retailing}$ $+ GWP_{rescue\ transportation}$
$GWP.IF_{animal\ feed}$	$GWP_{corn\ substitution}$ $+ GWP_{rescue\ transportation}$ $+ GWP_{transport\ to\ pig\ farm}$	$GWP_{corn\ substitution} + GWP_{retailing}$ $+ GWP_{rescue\ transportation}$ $+ GWP_{transport\ to\ pig\ farm}$
$GWP.IF_{AD}$	$GWP_{AD\ biogas\ to\ electricity}$ $+ GWP_{rescue\ transportation}$ $+ GWP_{transportation\ to\ facility}$	$GWP_{AD\ biogas\ to\ electricity} + GWP_{retailing}$ $+ GWP_{rescue\ transportation}$ $+ GWP_{transportation\ to\ facility}$
$GWP.IF_{Composting}$	$GWP_{food\ waste\ to\ compost}$ $+ GWP_{rescue\ transportation}$ $+ GWP_{transportation\ to\ facility}$	$GWP_{food\ waste\ to\ compost} + GWP_{retailing}$ $+ GWP_{rescue\ transportation}$ $+ GWP_{transportation\ to\ facility}$
$GWP.IF_{landfill}$	$GWP_{agriculture-adj.farm}$ $+ GWP_{landfilling}$ $+ GWP_{rescue\ transportation}$ $+ GWP_{transportation\ to\ landfill}$	$GWP_{agriculture-adj.farm} + GWP_{retailing}$ $+ GWP_{landfilling}$ $+ GWP_{rescue\ transportation}$ $+ GWP_{transportation\ to\ landfill}$

Units: kg CO_2e/lbs. **IF** $_{\textbf{agriculture-adj}}$ is adjusted for the weighted fraction of produce types donated by farms and retailers. GWP $_{\text{rescue transportation}}$ accounts for greenhouse gas emissions from donor–food bank–pantries transportation (Guo et al., 2023). **IF** $_{\textbf{retail}}$ includes storage at perishables distribution centers and supermarkets (Burek & Nutter, 2020). In **IF** $_{\textbf{AD}}$**,** AD stands for anaerobic digestion. For **IF** $_{\textbf{transportation to facility}}$, distances to composting and anaerobic digestion (AD) facilities are assumed to be the same to those of pig farms (2023).

References:

Burek, J., & Nutter, D. W. (2020). Environmental implications of perishables storage and retailing☆. *Renewable and Sustainable Energy Reviews*, *133*, 110070. https://doi.org/10.1016/j.rser.2020.110070

Guo, Z., Mu, T., Bozlak, C., Feingold, B., Hosler, A., Pettigrew, S., & Romeiko, X. X. (2023). *Comparing the Environmental Impacts of Representative Food Donation and Redistribution Strategies* (SSRN Scholarly Paper 4612106). https://doi.org/10.2139/ssrn.4612106
