# Supplementary material for: Sustainable Recovery from Shocks: Policies and Partnerships for Fresh Produce Rescue and Environmental Impact Reduction
Source: Foods. 2026 Feb 5;15(3):582. doi: 10.3390/foods15030582 (PMC12897177; doi:10.3390/foods15030582)
Supplement: Supplementary file 1 [file foods-15-00582-s001.zip › Supplementary File S2_COVID_Impacts_on_food_rescue_orgs_survey-corrected.pdf]

**Supplemental Information 2: COVID-19 Impacts on Food Rescue Organizations Survey:**

**Main Findings and Sample Questions**

**Table S1. Impacts of the COVID-19 shock on fresh produce rescue and food assistance organizations' responses in the New York Capital Region, findings from Stakeholder Survey (2021).**

| *Main themes* | *Subthemes* | *Description* |
|---|---|---|
| **Effects of the shock** | *Produce availability* | - Changes in fresh produce available to organizations and their clients; an initial drop in produce availability, followed by spikes of overabundant produce. |
| | *Demand for organizations' services* | - Increase in clients' visits and new clients. |
| | *Physical, economic, and human resources* | - More funds available to organizations to purchase food and through USDA and pandemic-created programs like Nourish New York.<br>- Reduced human capacity. Less staff and volunteers (*e.g.*, due to social distance measures and higher risk from COVID-19 of older volunteers). This included fewer available drivers and people to sort good from spoiled food. |
| **Organizations' responses** | *Operational changes* | - Changes to operational hours.<br>- Changes in deliveries of produce, handling, and storage. More contactless giveaway tables, mass distributions, customized bags, drop-offs to clients, and pre-packed items.<br>- Re-assignment of staff to produce sorting, packing, and distribution.<br>- Transition to paperless client application forms.<br>- While some organizations experienced loss of staff and volunteers, some managed to obtain more. |
| | *Organizations' coordination and partnerships* | - New partnerships and collaborations, including community projects, community gardens, and distribution through nonprofits.<br>- Increased and enhanced cooperation and communication among some organizations and local groups. However, communication was not always optimal, leading to potential inefficiencies and confusion. |

**Examples of questions that provided insights related to changes in fresh produce distribution**

- Have there been any federal, state, or local COVID-related policies that have impacted your produce-related activities or operations during COVID-19? (These could include an impact on either your handling of food, donation processes and/or amount, organization's ability to function, etc.)
    - Which policies have impacted your fresh produce-related activities or operations during COVID-19? (Examples: NOURISH NY, CARES Act, Pandemic EBT, specific municipal exemptions or lockdown measures, etc.)
    - How have policies impacted your fresh produce or waste management-related activities or operations during COVID-19?
- Have you observed any changes in fresh produce availability since March 7, 2020, that seem to be due to COVID-19 and not due to normal seasonal variation?
    - Please describe the changes you have seen in fresh produce availability since March 7, 2020.
- Which strategies have your organization implemented since March 7, 2020, to help avoid fresh produce waste?

<u>Examples of questions that provided insight related to changes in coordination and partnerships</u>:

- Please check the box next to the category of organizational policy change that your organization has had to make due to COVID-19. Please check all that apply.

    ☐ Operating hours.

    ☐ Eligibility for staff or volunteers.

    ☐ Eligibility criteria for organizations

☐ Eligibility criteria for clients.

☐ Volunteer policy changes.

(…)

Other. Please specify __________.

- Have there been any changes in the organizations receiving fresh produce donations from your organization since March 7, 2020 (*i.e.*, are you donating produce to different organizations than you were prior to COVID-19)?
    - Please describe the changes in the organizations (*e.g.*, type and or location of organization) receiving your produce donations since March 7, 2020.
- Since March 7, 2020, has your organization received fresh produce donations from different sources than what is typical for your organization?
    - Please describe the change in who is donating produce to your organization during COVID-19.
