# Supplementary material for: Sustainable Recovery from Shocks: Policies and Partnerships for Fresh Produce Rescue and Environmental Impact Reduction
Source: Foods. 2026 Feb 5;15(3):582. doi: 10.3390/foods15030582 (PMC12897177; doi:10.3390/foods15030582)
Supplement: Supplementary file 1 [file foods-15-00582-s001.zip › Supplementary File S3_Model_documentation_and_overview.pdf]

Supplemental Information 3: Model Documentation and Model Overview

Software: Vensim® DSS 10.1.5

File version: FoodRescue_Shocks_NutrAndEnv_3b_NYCR-Factors.mdl

Content:

- S3a: Model Documentation (equations)
- S3b: Model Overview (model diagram)

S3a: Model Documentation

```
Average time to consumption=
        1
    Units: Weeks
    Assuming pantry users would consume the food over one
week.

CARBON IMPACT FACTOR FOR COMPOSTED FARM FOOD=
    0.0206
Units: kgCO2e/Pound

CARBON IMPACT FACTOR FOR COMPOSTED RETAIL FOOD=
    0.0455
Units: kgCO2e/Pound

CARBON IMPACT FACTOR FOR DONATED FARM FOOD=
    0.0099
Units: kgCO2e/Pound
CARBON FOOTPRINT Impact factor of donated farm produce.

CARBON IMPACT FACTOR FOR DONATED RETAIL FOOD=
    0.0324
Units: kgCO2e/Pound

CARBON IMPACT FACTOR FOR WASTE FROM RETAIL TO LANDFILL=
    0.1678
Units: kgCO2e/Pound
CARBON FOOTPRINT Impact factor (upstream and downstream
carbon emissions) of wasted fresh produce from retail and
sent to LANDFILL.

CARBON IMPACT FACTOR FOR WASTED FARM FOOD TO AD=
    -0.0227
Units: kgCO2e/Pound

CARBON IMPACT FACTOR FOR WASTED FARM PRODUCE TO LANDFILL=
    0.1407
Units: kgCO2e/Pound
CARBON FOOTPRINT Impact factor (upstream and downstream
carbon emissions) of wasted fresh produce FROM FARMS and
sent to LANDFILL.

CARBON IMPACT FACTOR FOR WASTED RETAIL FOOD TO AD=
    0.0021
Units: kgCO2e/Pound

composting to AD ratio=
    0.5
```

Units: Dmnl

Donated Produce at Households= INTEG (
    produce distribution to households-produce consumed by households-household waste rate,
        91285.7
)
Units: Pounds

farm to retail ratio=
    surplus from growers/"non-farm surplus"
Units: Dmnl
surplus from growers with respect to surplus from retail
        (non-farms)

GHGs from composted waste=
    farm to retail ratio*(total waste rate from organizations*WASTE FRACTION TO COMPOSTING
*CARBON IMPACT FACTOR FOR COMPOSTED FARM FOOD)+
    (1-farm to retail ratio)*(total waste rate from organizations*WASTE FRACTION TO COMPOSTING
*CARBON IMPACT FACTOR FOR COMPOSTED RETAIL FOOD)
Units: kgCO2e/Week

GHGs from donated food=
    farm to retail ratio*produce consumed by households*CARBON IMPACT FACTOR FOR DONATED FARM FOOD
+
    (1-farm to retail ratio)*produce consumed by households*CARBON IMPACT FACTOR FOR DONATED RETAIL FOOD
Units: kgCO2e/Week
GHG emissions in MTCO2e of fresh produce successfully
        distributed (donated).
GHGs from landfilled waste=
    farm to retail ratio*(total waste rate from organizations*WASTE FRACTION TO LANDFILL
*CARBON IMPACT FACTOR FOR WASTED FARM PRODUCE TO LANDFILL
) +
    (1-farm to retail ratio)*(total waste rate from organizations*WASTE FRACTION TO LANDFILL
*CARBON IMPACT FACTOR FOR WASTE FROM RETAIL TO LANDFILL
) +
    household waste rate*0.1249
Units: kgCO2e/Week
GHG emissions in MTCO2e generated from food wasted at organizations and sent to landfill. This does not account for additional transportation and impacts from farm to organizations nor emissions at food rescue organizations. It serves the purpose of policy comparison because emissions at organizations can be assumed to be the same regardless of the origin of food. 0.1249 is the impact factor for landfilled produce that already went through the donation process.

GHGs from waste used in AD=
    farm to retail ratio*(total waste rate from organizations*WASTE FRACTION TO AD
*CARBON IMPACT FACTOR FOR WASTED FARM FOOD TO AD)+
    (1-farm to retail ratio)*(total waste rate from organizations*WASTE FRACTION TO AD
*CARBON IMPACT FACTOR FOR WASTED RETAIL FOOD TO AD)
Units: kgCO2e/Week

GWP from waste used as animal feed=
    farm to retail ratio*(total waste rate from organizations*WASTE FRACTION TO ANIMAL FEED
*IMPACT FACTOR OF FARM PRODUCE TO ANIMAL FEED)+
    (1-farm to retail ratio)*(total waste rate from organizations*WASTE FRACTION TO ANIMAL FEED
*IMPACT FACTOR OF RETAIL PRODUCE TO ANIMAL FEED)
Units: kgCO2e/Week
GHG emissions in MTCO2e generated from food wasted at organizations and sent to Animal Feed (e.g., pig farms). This does not account for additional transportation and impacts from farm to organizations nor emissions at food rescue organizations. It serves the purpose of policy comparison because emissions at organizations can be assumed to be the same regardless of the origin of food.

GWP Stock from Composted Produce= INTEG (
    GHGs from composted waste,
        0)
Units: kgCO2e
Global warming potential from wasted food used as compost.

GWP Stock from Donated Produce= INTEG (

```
    GHGs from donated food,
        0)
Units: kgCO2e

GWP Stock from Food Used in AD= INTEG (
    GHGs from waste used in AD,
        0)
Units: kgCO2e

GWP Stock from Landfilled Produce= INTEG (
    GHGs from landfilled waste,
        0)
Units: kgCO2e
GWP - GHG emissions in CO2e

GWP Stock from Produce Waste Used as Animal Feed= INTEG (
    GWP from waste used as animal feed,
        0)
Units: kgCO2e

household waste fraction=
    0.2
Units: Dmnl
Rough assumption based on Muth et al. 2011 (USDA Economic
Research Service Report) and Sundin et al. (2022). Also,
assuming that food acquired by households and not consumed
will end up in landfills.
     Based on a study on household waste in the U.S.
(Muth et al., 2011), and a study on donated surplus in
Sweden (Sundin et al., 2022), 20% to 25% of food donated
to households might be wasted. We assumed the level of
waste at the lower end, which might overestimate the
benefits of donation.


household waste rate=
    Donated Produce at Households*household waste
fraction/Average time to consumption
Units: Pounds/Week

IMPACT FACTOR OF FARM PRODUCE TO ANIMAL FEED=
    -0.0051
Units: kgCO2e/Pound
CARBON FOOTPRINT Impact factor (upstream and downstream
carbon emissions) of wasted fresh produce FROM FARMS and
diverted to animal feed.

IMPACT FACTOR OF RETAIL PRODUCE TO ANIMAL FEED=
    0.0197
Units: kgCO2e/Pound
CARBON FOOTPRINT Impact factor (upstream and downstream
carbon emissions) of wasted fresh produce FROM RETAIL and
diverted to animal feed.

"non-farm surplus"=
    SURPLUS FROM RETAIL RATE
Units: Pounds/Week

NORMAL WASTE FRACTION TO LANDFILL=
    0.4
Units: Dmnl

produce consumed by households=
    Donated Produce at Households/Average time to
consumption*(1-household waste fraction
)
Units: Pounds/Week

produce distribution to households=
    Fresh Produce at Pantries/TIME TO MAKE PRODUCE
AVAILABLE AT PANTRIES
Units: Pounds/Week

RECYCLE FOOD USUALLY GOING TO LANDFILL=
    1
Units: Dmnl [0,1]

surplus from growers=
    SURPLUS FROM GROWERS RATE
Units: Pounds/Week

Total GWP=
    GWP Stock from Donated Produce +
    GWP Stock from Produce Waste Used as Animal Feed +
    GWP Stock from Landfilled Produce +
    GWP Stock from Composted Produce +
```

```
    GWP Stock from Food Used in AD
Units: kgCO2e

total waste rate from organizations=
    wasted produce at early stage+wasted produce at
pantries
Units: Pound/Week

Total Water Footprint=
    Water Footprint from Donated Produce +
    Water Footprint from Produce Used as Animal Feed +
    Water Footprint from Landfilled Produce +
    Water Footprint from Composted Produce +
    Water Footprint from Food Used in Anaerobic Digestion
Units: gallons

WASTE FRACTION TO AD=
    IF THEN ELSE(
       RECYCLE FOOD USUALLY GOING TO LANDFILL = 1 ,
       NORMAL WASTE FRACTION TO LANDFILL*(1-composting to
AD ratio) , 0 )
Units: Dmnl

WASTE FRACTION TO ANIMAL FEED=
    0.6
Units: Dmnl
Assumption based on partner's data.

WASTE FRACTION TO COMPOSTING=
    IF THEN ELSE(
       RECYCLE FOOD USUALLY GOING TO LANDFILL = 1 ,
       NORMAL WASTE FRACTION TO LANDFILL*composting to AD
ratio , 0 )
Units: Dmnl
The wasted fraction is divided by 2 because we assume that
the
        other half goes to anaerobic digestion (AD)

WASTE FRACTION TO LANDFILL=
    IF THEN ELSE(
       RECYCLE FOOD USUALLY GOING TO LANDFILL = 1 ,
       0, NORMAL WASTE FRACTION TO LANDFILL )
Units: Dmnl

wasted produce at early stage=
    maximum outflow at early stage*(wasted fraction at
early stage)
Units: Pounds/Week

wasted produce at pantries=
    produce distribution to households*wasted fraction at
pantries
Units: Pounds/Week

Water Footprint from Composted Produce= INTEG (
    water from composted waste,
        0)
Units: gallons

Water Footprint from Donated Produce= INTEG (
    water in donated food,
        0)
Units: gallons

Water Footprint from Food Used in Anaerobic Digestion=
INTEG (
    water from waste used in AD,
        0)
Units: gallons

Water Footprint from Landfilled Produce= INTEG (
    water from landfilled waste,
        0)
Units: gallons

Water Footprint from Produce Used as Animal Feed= INTEG (
    water from waste used as animal feed,
        0)
Units: gallons

water from composted waste=
    farm to retail ratio*(total waste rate from
organizations*WASTE FRACTION TO COMPOSTING
*WATER IMPACT FACTOR FOR COMPOSTED FARM FOOD)+
    (1-farm to retail ratio)*(total waste rate from
organizations*WASTE FRACTION TO COMPOSTING
```

```
*WATER IMPACT FACTOR FOR COMPOSTED RETAIL FOOD)
Units: gallons/Week

water from landfilled waste=
    farm to retail ratio*(total waste rate from
organizations*WASTE FRACTION TO LANDFILL
*WATER IMPACT FACTOR FOR WASTED FARM PRODUCE TO LANDFILL)
+
    (1-farm to retail ratio)*(total waste rate from
organizations*WASTE FRACTION TO LANDFILL
*WATER IMPACT FACTOR FOR WASTE FROM RETAIL TO LANDFILL)
Units: gallons/Week

water from waste used as animal feed=
    farm to retail ratio*(total waste rate from
organizations*WASTE FRACTION TO ANIMAL FEED
*WATER IMPACT FACTOR FOR WASTED FARM PRODUCE TO ANIMAL
FEED)+
    (1-farm to retail ratio)*(total waste rate from
organizations*WASTE FRACTION TO ANIMAL FEED
*WATER IMPACT FACTOR FOR WASTED RETAIL PRODUCE TO ANIMAL
FEED)
Units: gallons/Week

water from waste used in AD=
    farm to retail ratio*(total waste rate from
organizations*WASTE FRACTION TO AD
*WATER IMPACT FACTOR FOR WASTED FARM FOOD TO AD) +
    (1-farm to retail ratio)*(total waste rate from
organizations*WASTE FRACTION TO AD
*WATER IMPACT FACTOR FOR WASTED RETAIL FOOD TO AD)
Units: gallons/Week

WATER IMPACT FACTOR FOR COMPOSTED FARM FOOD=
    16.124
Units: gallons/Pound

WATER IMPACT FACTOR FOR COMPOSTED RETAIL FOOD=
    20.122
Units: gallons/Pound

WATER IMPACT FACTOR FOR DONATED FARM PRODUCE=
    8.06
Units: gallons/Pound

WATER IMPACT FACTOR FOR DONATED RETAIL PRODUCE=
    10.061
Units: gallons/Pound

WATER IMPACT FACTOR FOR WASTE FROM RETAIL TO LANDFILL=
    20.122
Units: gallons/Pound

WATER IMPACT FACTOR FOR WASTED FARM FOOD TO AD=
    16.124
Units: gallons/Pound

WATER IMPACT FACTOR FOR WASTED FARM PRODUCE TO ANIMAL
FEED=
    16.124
Units: gallons/Pound

WATER IMPACT FACTOR FOR WASTED FARM PRODUCE TO LANDFILL=
    16.124
Units: gallons/Pound

WATER IMPACT FACTOR FOR WASTED RETAIL FOOD TO AD=
    20.122
Units: gallons/Pound

WATER IMPACT FACTOR FOR WASTED RETAIL PRODUCE TO ANIMAL
FEED=
    20.122
Units: gallons/Pound

water in donated food=
    farm to retail ratio*produce consumed by
households*WATER IMPACT FACTOR FOR DONATED FARM PRODUCE
 +
    (1-farm to retail ratio)*produce consumed by
households*WATER IMPACT FACTOR FOR DONATED RETAIL PRODUCE
Units: gallons/Week
```

S3b: Model Overview

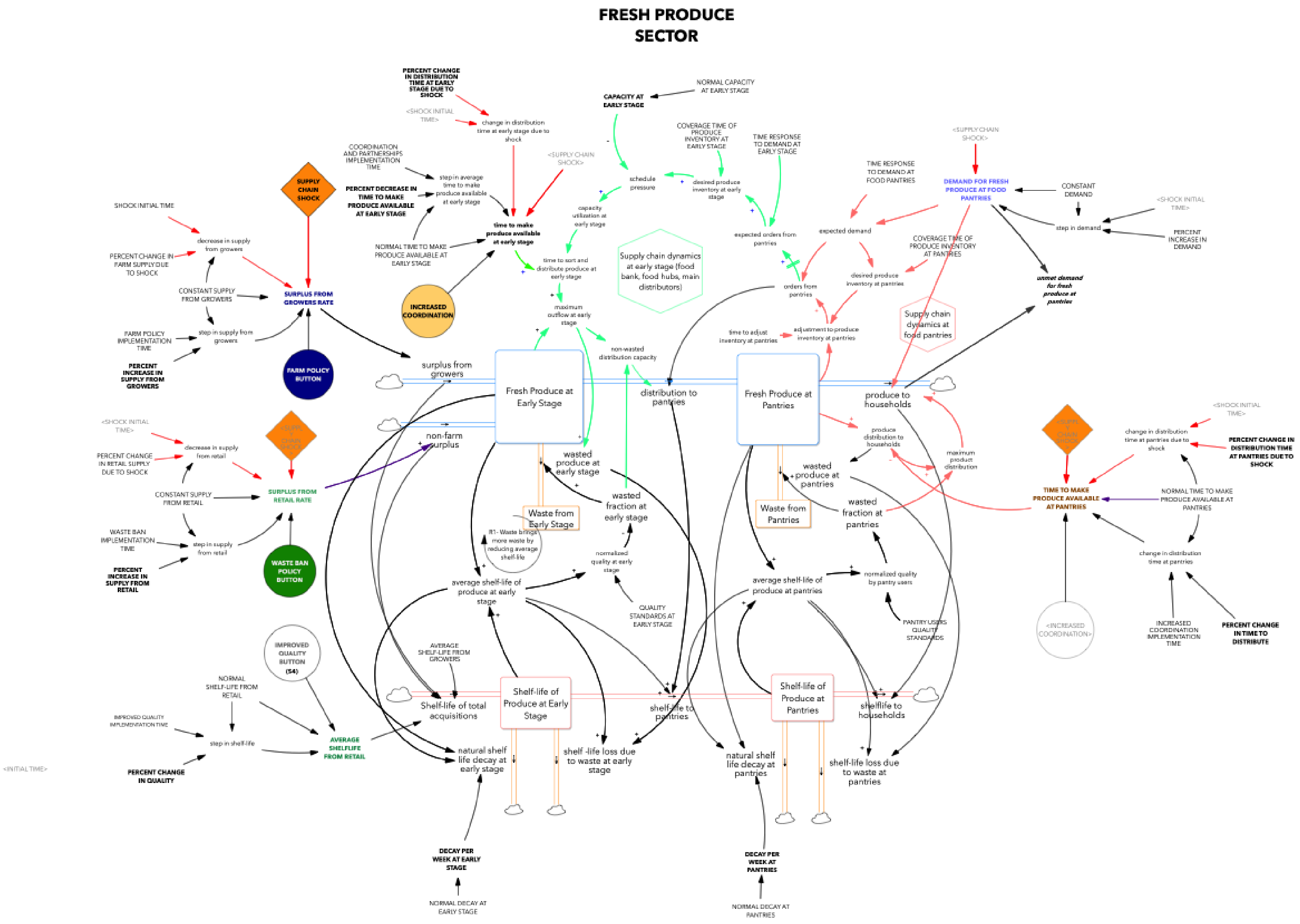

ENVIRONMENTAL SECTOR

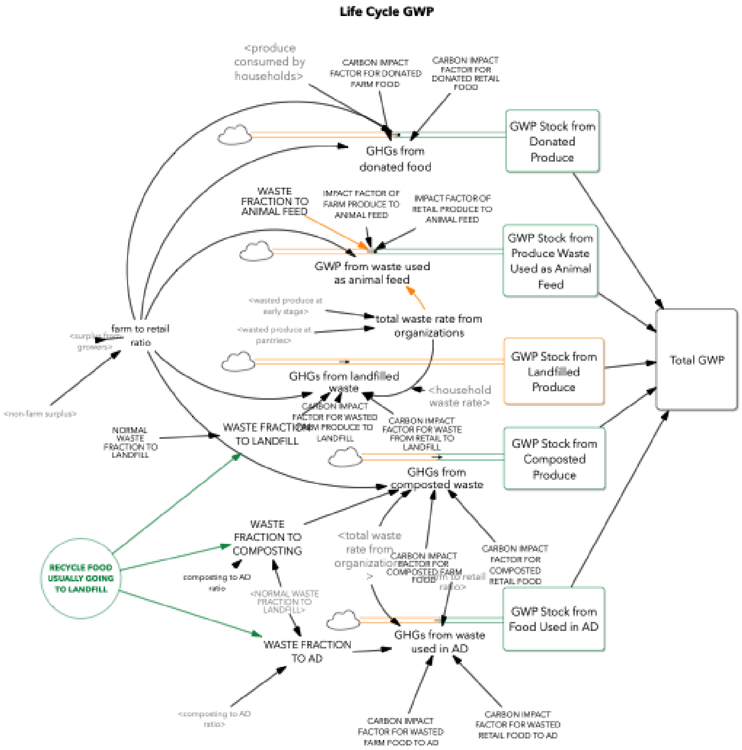

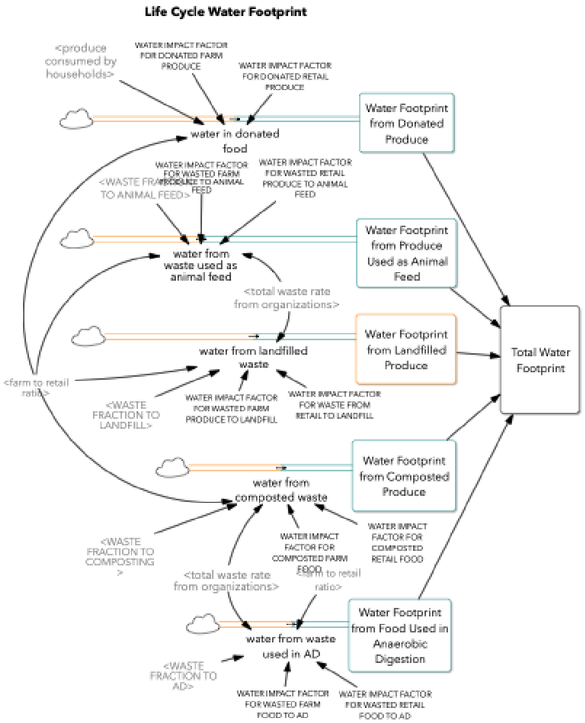

HOUSEHOLD SECTOR

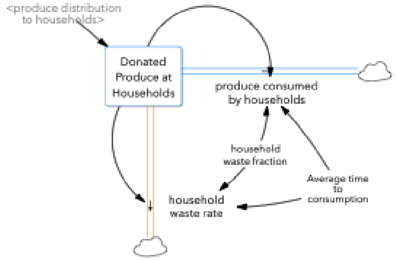

Go to top
